# Supplementary material for: Repression of germline genes by PRC1.6 and SETDB1 in the early embryo precedes DNA methylation-mediated silencing
Source: Nat Commun. 2021 Dec 2;12:7020. doi: 10.1038/s41467-021-27345-x (PMC8639735; doi:10.1038/s41467-021-27345-x)
Supplement: Supplementary file 1 — Supplementary information. [file 41467_2021_27345_MOESM1_ESM.pdf]

Supplementary Information

Repression of germline genes by PRC1.6 and SETDB1 in the early embryo precedes DNA methylation-mediated silencing

Mochizuki K et al.

8 Supplementary Figures

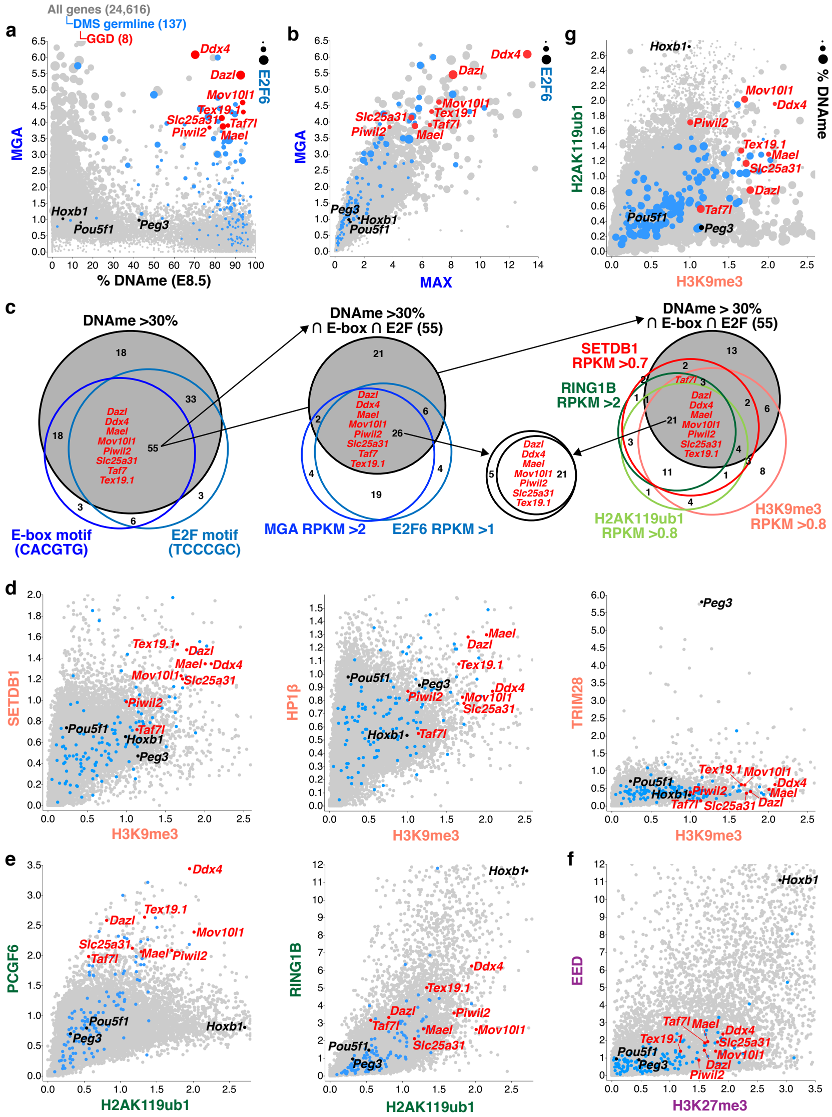

Supplementary Figure 1. Enrichment of repressor complex subunits and associated epigenetic marks at DMS germline genes in pESCs. Related to Fig. 1.

(**a**) Scatterplot showing the relationship between the enrichment of MGA in pESCs or E2F6 in nESCs and % DNAme (TSS -0.9/+0.4 kb) in E8.5 embryos. (**b**) Scatterplot showing the relationship between the enrichment (RPKM) of MGA, MAX and E2F6 in pESCs in genic TSS regions (+/-2 kb). (**c**) Left Venn diagram illustrates the overlap between the 124 (of 137 total) DMS germline genes showing >30% DNAme in the TSS region (-0.9/+0.4 kb) in pESCs with the presence of an E-box and/or E2F consensus motif and reveal that all GGD genes are included among the 55 genes with both motifs. Middle and right Venn diagram illustrate the intersections (∩) between the 55 DMS germline genes which show >30% DNAme in the TSS region (-0.9/+0.4 kb) in pESCs and have both E-box and E2F consensus motifs with bona fide MGA (RPKM >2) and E2F6 (RPKM >1) enrichment

and SETDB1 (RPKM >0.7), H3K9me3 (RPKM >0.8), RING1B (RPKM >2) and/or H2AK119ub1 (RPKM >0.8) enrichment, respectively, in the TSS region (TSS +/-2 kb), as measured by ChIPseq in ESCs. Almost all GGD genes (7/8) are included among the 21 genes enriched for MGA, E2F6, SETDB1, H3K9me3, RING1B, and H2AK119ub1 in ESCs. (**d-f**) Scatterplots showing the relationship between the enrichment of (**d**) repressor complex II or (**e-f**) repressor complex III subunits or associated histone PTMs in genic TSS regions (+/-2 kb). (**g**) Scatterplot showing the relationship between H2AK119ub1 and H3K9me3 enrichment, with the % DNAme (TSS -0.9/+0.4 kb) depicted by dot size. All ChIP-seq data are presented as RPKM values.

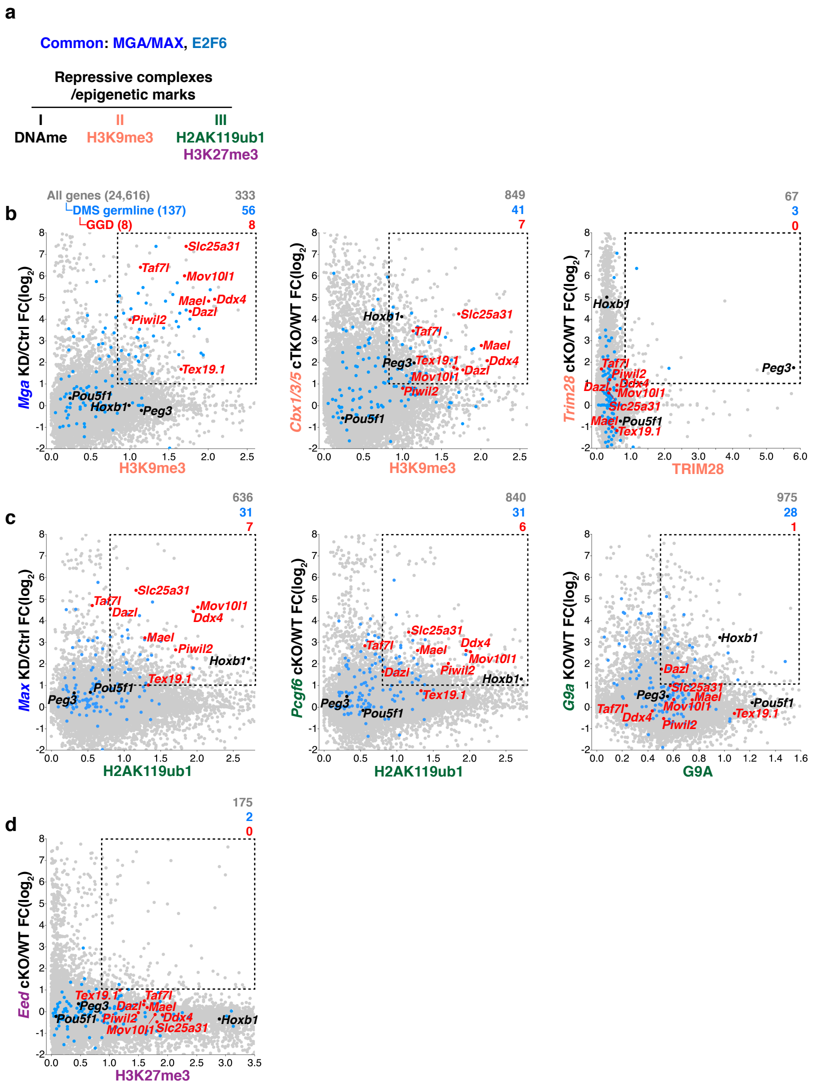

Supplementary Figure 2. Impact of depletion of key subunits of previously implicated repressive complexes on expression of DMS germline genes in pESCs. Related to Fig. 2.

(**a**) Color codes for repressive complex subunits or the DNA/chromatin marks they deposit shown in panels **b-d**. (**b-d**) Scatterplots showing the fold-change (FC) of gene expression in pESCs following KO or KD of common factors or repressor complex subunits (y-axis) versus enrichment (RPKM) of relevant histone PTMs/subunits (TSS +/-2 kb) (x-axis). For each category of genes (color-coded as shown in panel b), the total number of genes is shown in parentheses. The number showing a >2-fold increase in expression and ChIPseq enrichment (RPKM >0.8 or >0.5), is also shown at the top right of each plot.

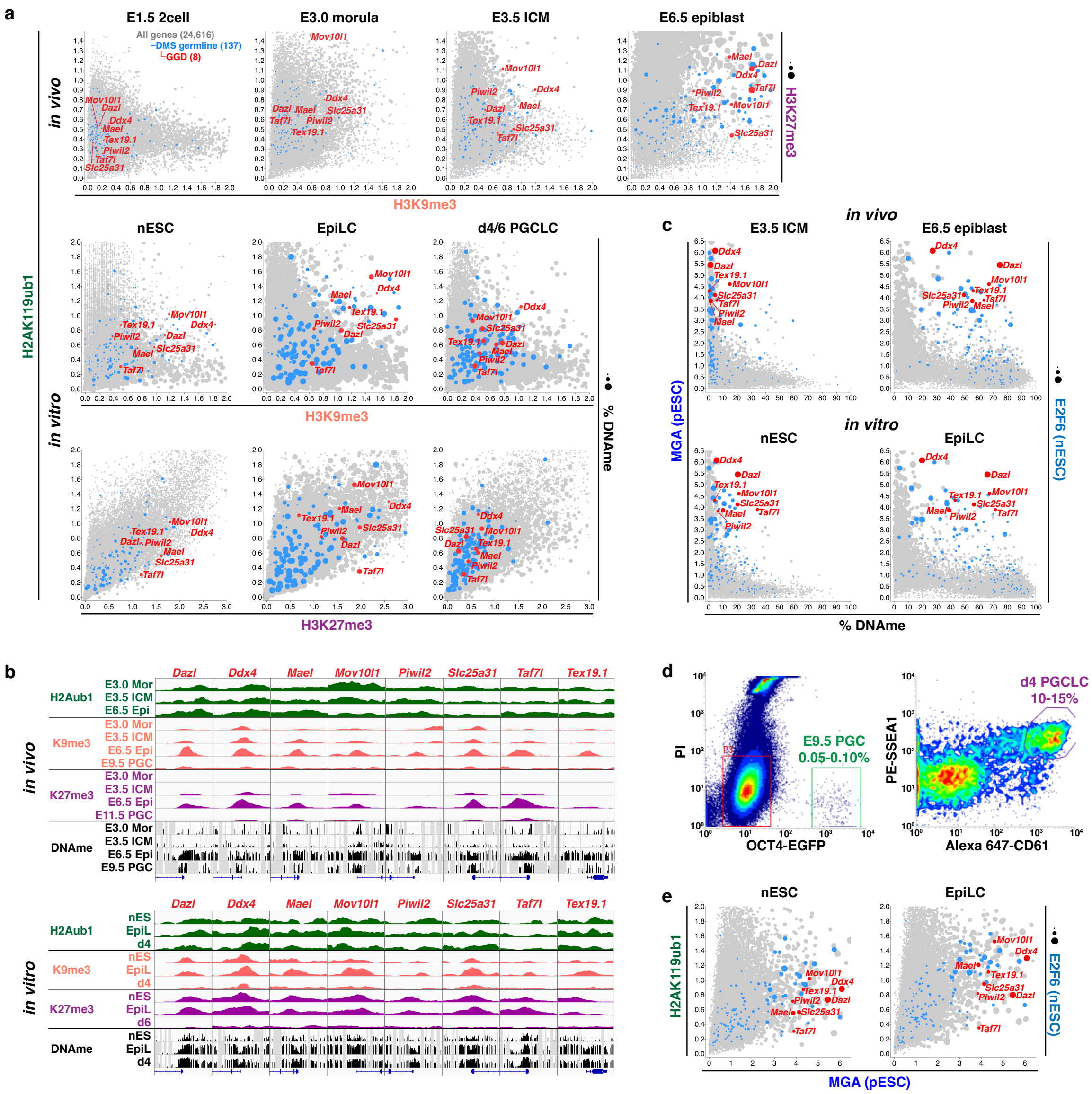

Supplementary Figure 3. GGD genes are hypomethylated and enriched for H2AK119ub1, H3K9me3 and H3K27me3 in the pre-implantation embryo and nESCs.

(**a**) (Top) Scatterplots showing the relationship between the enrichment (RPKM) of H2AK119ub1 and H3K9me3 during early embryonic development in regions flanking all genic promoters (TSS +/-2 kb). The range of enrichment levels (RPKM) of H3K27me3 over the same developmental stages is depicted by dot size. (Bottom) Scatterplots showing the relationship between the enrichment (RPKM) of H2AK119ub1 and H3K9me3 or H3K27me3 in nESCs, EpiLCs and d4/6 PGCLCs at all genic promoters (TSS +/-2 kb). The range of % DNAme in the promoter region (TSS -0.9 kb/+0.4 kb) over the same developmental stages is depicted by dot size. All genes, DMS germline genes and GGD genes are color-coded as shown. (**b**) Genome browser track showing RPM values of

H2AK119ub1, H3K9me3 and H3K27me3 data as well as % DNAme in the promoter regions (TSS+/-3kb) of GGD genes at the developmental time points shown. For each WGBS track, regions highlighted in grey reflect the absence of DNAme data. (**c**) Scatterplots showing the relationship between the enrichment (RPKM) of MGA in pESCs and % DNAme (TSS -0.9/+0.4 kb) at the developmental time points shown. E2F6 enrichment (in nESCs) is depicted by dot size (RPKM). (**d**) FACS sorting strategy for purification of E9.5 PGCs and d4 PGCLCs using OCT4-EGFP and PE-SSEA1/Alexa 647-CD61, respectively. For E9.5 PGCs, dead cells were excluded by gating out PI positive cells during FACS. Sorted PGCs (0.05-0.10% of total hindgut endoderm cells) and PGCLCs (10-15% of total cells in aggregates) were processed for ChIP-seq. (**e**) Scatterplots showing the relationship between the enrichment (RPKM) of MGA (in pESCs) and H2AK119ub1 in nESCs and EpiLCs. E2F6 enrichment (in nESCs) is depicted by dot size (RPKM).

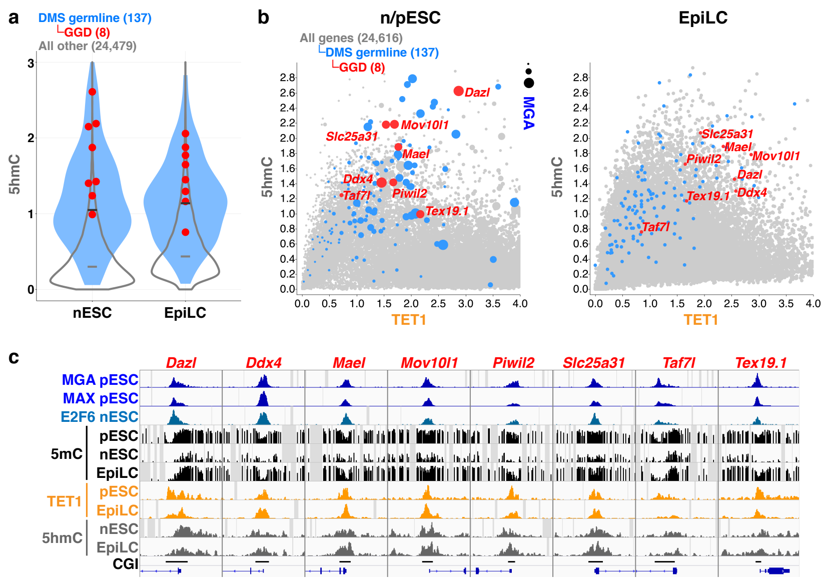

Supplementary Figure 4. DMS germline genes are enriched for TET1 and 5hmC in nESCs and EpiLCs. Related to Fig. 4.

(**a**) Violin plots showing 5hmC (RPKM) profiles in regions flanking the promoters (TSSs +/-1 kb) of the 137 DMS germline (blue filled), 8 GGD (red data points) and all other genes (open) in nESCs and EpiLCs. (**b**) Scatterplots showing the relationship between the enrichment (RPKM) of TET1 in pESCs or EpiLCs versus 5hmC in nESCs and EpiLCs in the promoter region (TSS +/-1 kb). The range of the enrichment (RPKM) of MGA (in pESCs) is depicted by dot size. All, DMS germline and GGD genes are color-coded as shown. (**c**) Genome browser tracks of the promoter regions (TSS +/-3 kb) of GGD genes showing ChIP-seq, WGBS and 5hmCIP-seq in the indicated cells. RPM values are shown for MGA, MAX, E2F6, TET1 and 5hmC and % 5mC for DNAme. For each WGBS track, regions highlighted in grey reflect the absence of DNAme data.

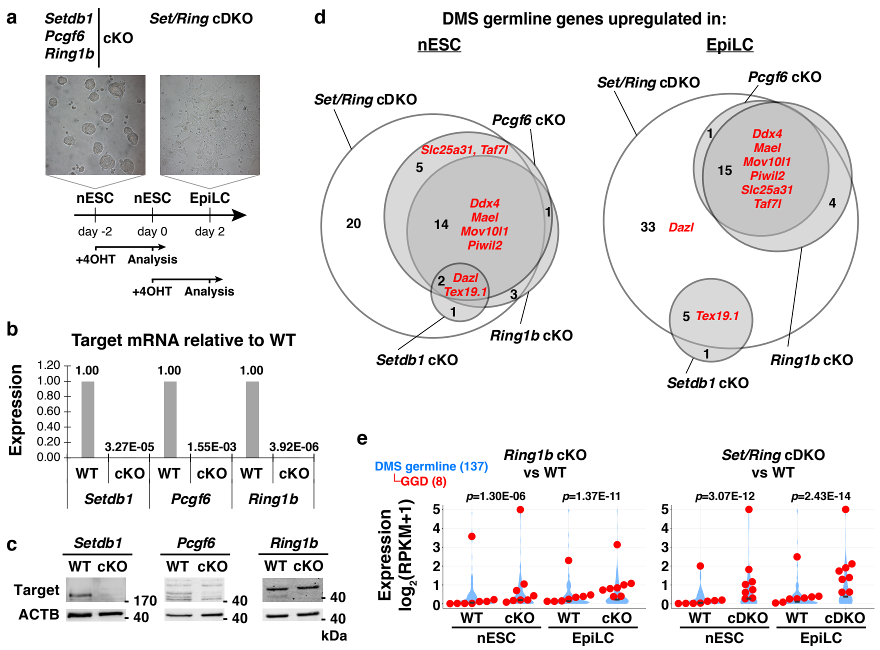

Supplementary Figure 5. Redundant roles of SETDB1 and RING1B in PRC1.6-mediated silencing of DMS germline genes in nESCs and EpiLCs. Related to Fig. 5.

(**a**) A schematic representation of the timing of 4OHT-induction of *Setdb1*, *Pcgf6*, *Ring1b* or *Setdb1* and *Ring1b* (*Set*/*Ring)* deletion and harvest of nESCs and EpiLCs. (**b**) Deletion efficiency of *Setdb1*, *Pcgf6* and *Ring1b* in *Setdb1*, *Pcgf6* and *Ring1b* cKO EpiLCs, respectively, as measured by qRT-PCR. *Arbp* was used as an internal control. (**c**) Western analysis of SETDB1, PCGF6 and RING1B protein levels (arrows) in *Setdb1*, *Pcgf6* and *Ring1b* cKO EpiLCs, respectively. EpiLCs were harvested at day 2 post 4OHT. β-actin (ACTB) was used as an internal control. Representative images from two independent experiments are shown. Original blots are provided as a Source Data file. (**d**) Venn diagrams showing the overlap among DMS germline genes upregulated in nESCs or EpiLCs following cKO of the indicated chromatin factors. (**e**) Violin plots showing gene expression of the 137 DMS germline (blue filled) and 8 GGD genes (red data points) in control (WT), *Ring1b* cKO or *Set*/*Ring* cDKO nESCs and EpiLCs (n=2 for each cKO and parent line). Two-sided paired-samples *t*-tests were performed for each WT/KO pair of all germline gene values.

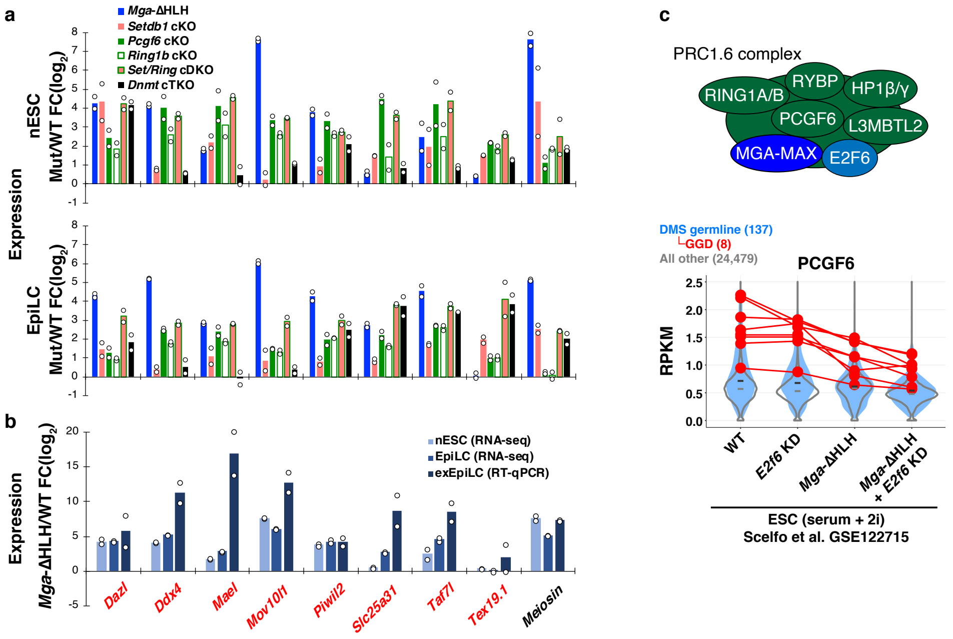

Supplementary Figure 6. MGA inhibits expression of GGD genes via PRC1.6 dependent and independent mechanisms. Related to Fig. 5.

(**a**) Bar graph showing the mean FC ($\log_2$) in expression of GGD genes and *Meiosin* in nESCs and EpiLCs for each of the KO lines indicated. Data points show biological duplicates. (**b**) Bar graph showing the mean FC ($\log_2$) in expression of GGD genes and *Meiosin* in *Mga*-ΔHLH mutant versus WT nESCs, EpiLCs and exEpiLCs. Data points show biological duplicates. (**c**) Violin plots showing PCGF6 enrichment (RPKM) at the TSS (+/-2 kb) of 137 DMS germline (filled), 8 GGD (red data points) and all other gene loci (open) in control (WT) versus *E2f6* KD, *Mga*-ΔHLH or *Mga*-ΔHLH + *E2f6* KD ESCs (in serum + 2i culture)[1].

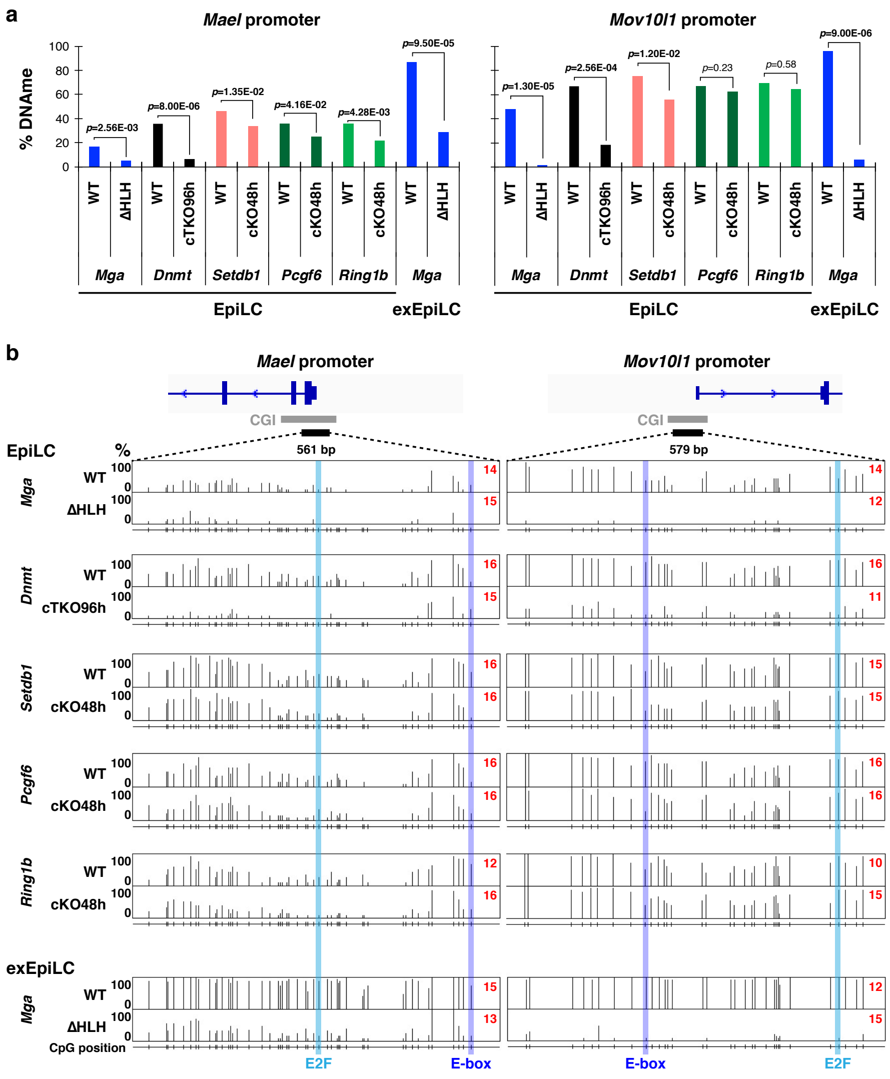

Supplementary Figure 7. Role of MGA, SETDB1 and/or PRC1.6 in DNAme of GGD genes in EpiLCs. Related to Fig. 5.

(**a**) Bar graphs showing the mean levels of DNAme, as determined by Sanger bisulphite sequencing, in the promoter regions of GGD genes *Mael* and *Mov10l1* in control (WT) versus *Mga*-ΔHLH, *Dnmt* cTKO, *Setdb1* cKO, *Pcgf6* cKO or *Ring1b* cKO EpiLCs. Data from exEpiLCs is also shown for WT vs *Mga*-ΔHLH. Two-sided Mann-Whitney U-tests were performed between each mutant and WT. For *Setdb1*, *Pcgf6* and *Ring1b* lines, cultures were harvested at day 2 post 4OHT (cKO48h). (**b**) DNAme profiles of the CGI promoter regions of *Mael* and *Mov10l1* in WT and *Mga*-ΔHLH EpiLCs/exEpiLCs and *Setdb1* cKO, *Pcgf6* cKO or *Ring1b* cKO EpiLCs. The number of molecules sequenced for each data set is shown in red and the mean DNAme level at each CpG is shown. E-box and E2F motifs in each locus are also shown.

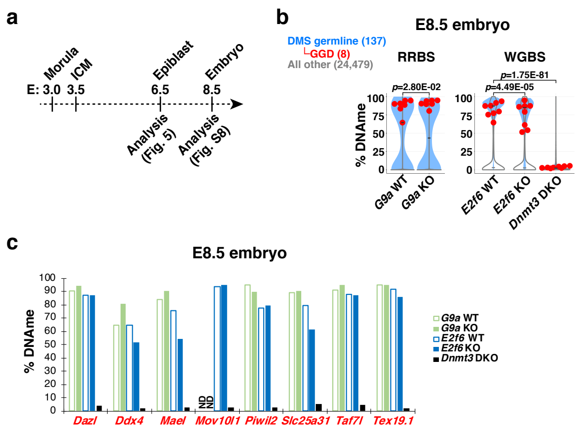

Supplementary Figure 8. Role of G9A or E2F6 in DNAme of DMS germline genes in E8.5 embryos. Related to Fig. 5.

(**a**) A schematic representation of early embryonic development. (**b**) Violin plots showing DNAme profiles of the promoter regions (TSS +/-0.3 kb) of 137 DMS germline (filled), 8 GGD (red data points) and all other gene loci (open) in WT, *G9a*- or *E2f6* KO E8.5 embryos. Two-sided paired-sample *t*-tests were performed for each KO and WT pair of all DMS germline gene values. (**c**) Bar graph showing the mean levels of DNAme in the promoter regions (TSS +/-0.3 kb) of GGD genes in WT versus *G9a- or E2f6* KO E8.5 embryos, as measured by RRBS[2] or WGBS[3]. DNAme levels in *Dnmt3* DKO E8.5 embryos is also shown for comparison.

Supplementary references

1. Scelfo, A. *et al.* Functional Landscape of PCGF Proteins Reveals Both RING1A/B-Dependent- and RING1A/B- Independent-Specific Activities. *Mol. Cell* **74,** 1037–1052.e7 (2019).
2. Auclair, G. *et al.* EHMT2 directs DNA methylation for efficient gene silencing in mouse embryos. *Genome Res.* **26,** 192–202 (2016).
3. Dahlet, T. *et al.* E2F6 initiates stable epigenetic silencing of germline genes during embryonic development. *Nature Communications* 1–14 (2021). doi:10.1038/s41467-021-23596-w
