# Supplementary material for: Repression of germline genes by PRC1.6 and SETDB1 in the early embryo precedes DNA methylation-mediated silencing
Source: Nat Commun. 2021 Dec 2;12:7020. doi: 10.1038/s41467-021-27345-x (PMC8639735; doi:10.1038/s41467-021-27345-x)
Supplement: Supplementary file 3 — Description of Additional Supplementary Files. [file 41467_2021_27345_MOESM3_ESM.pdf]

Description of Additional Supplementary Files

File Name: Supplementary Data 1
Description: List of 137 DNA methylation sensitive (DMS) germline genes identified Dahlet et al. (2020).

File Name: Supplementary Data 2
Description: List of previously published datasets used in this study.

File Name: Supplementary Data 3
Description: List of 40 DMS germline genes bound by MGA (RPKM >2) & E2F6 (RPKM >1) and enriched for DNAme (% >30) in ESCs. Related to Figure 1b.

File Name: Supplementary Data 4
Description: Lists of germline genes significantly upregulated and top 5 GO terms among the terms associated with genes significantly upregulated in the indicated mutant cells.

File Name: Supplementary Data 5
Description: Oligonucleotide sequences used in this study.

File Name: Supplementary Data 6
Description: Summary of ChIP-seq and RNA-seq generated in this study.
