# Supplementary material for: Repression of germline genes by PRC1.6 and SETDB1 in the early embryo precedes DNA methylation-mediated silencing
Source: Nat Commun. 2021 Dec 2;12:7020. doi: 10.1038/s41467-021-27345-x (PMC8639735; doi:10.1038/s41467-021-27345-x)
Supplement: Supplementary file 10 — Reporting summary. [file 41467_2021_27345_MOESM10_ESM.pdf]

nature research

Corresponding author(s): Matthew Lorincz

Last updated by author(s): Oct 23, 2021

Reporting Summary

Nature Research wishes to improve the reproducibility of the work that we publish. This form provides structure for consistency and transparency in reporting. For further information on Nature Research policies, see Authors & Referees and the Editorial Policy Checklist.

Statistics

For all statistical analyses, confirm that the following items are present in the figure legend, table legend, main text, or Methods section.

n/a	Confirmed	
☐	☒	The exact sample size (*n*) for each experimental group/condition, given as a discrete number and unit of measurement
☐	☒	A statement on whether measurements were taken from distinct samples or whether the same sample was measured repeatedly
☐	☒	The statistical test(s) used AND whether they are one- or two-sided<br>*Only common tests should be described solely by name; describe more complex techniques in the Methods section.*
☒	☐	A description of all covariates tested
☐	☒	A description of any assumptions or corrections, such as tests of normality and adjustment for multiple comparisons
☐	☒	A full description of the statistical parameters including central tendency (e.g. means) or other basic estimates (e.g. regression coefficient) AND variation (e.g. standard deviation) or associated estimates of uncertainty (e.g. confidence intervals)
☐	☒	For null hypothesis testing, the test statistic (e.g. *F*, *t*, *r*) with confidence intervals, effect sizes, degrees of freedom and *P* value noted<br>*Give P values as exact values whenever suitable.*
☒	☐	For Bayesian analysis, information on the choice of priors and Markov chain Monte Carlo settings
☒	☐	For hierarchical and complex designs, identification of the appropriate level for tests and full reporting of outcomes
☒	☐	Estimates of effect sizes (e.g. Cohen's *d*, Pearson's *r*), indicating how they were calculated

Our web collection on statistics for biologists contains articles on many of the points above.

Software and code

Policy information about availability of computer code

Data collection	Softwares installed on Illumina's sequencers (NextSeq 500, NextSeq 2000 and HiSeq 2000) were used for data collection.
Data analysis	bedtools 2.22.1<br>bwa 0.7.10<br>cutadapt 1.15<br>DESeq2 1.30.0<br>FastQC v0.11.2<br>IGV 2.3.98<br>Microsoft Excel 16.47.1<br>picard-tools 1.128<br>R 4.0.0<br>RSEM 1.3.0<br>samtools 1.1<br>sratoolkit.2.5.0<br>STAR 2.5.3a<br>TCC-GUI 2021.01.26<br>TrimGalore 0.4.5<br>VisR 0.9.41

For manuscripts utilizing custom algorithms or software that are central to the research but not yet described in published literature, software must be made available to editors/reviewers. We strongly encourage code deposition in a community repository (e.g. GitHub). See the Nature Research guidelines for submitting code & software for further information.

Data

Policy information about availability of data

All manuscripts must include a data availability statement. This statement should provide the following information, where applicable:

- Accession codes, unique identifiers, or web links for publicly available datasets
- A list of figures that have associated raw data
- A description of any restrictions on data availability

ChIP-seq and RNA-seq data generated in this study have been deposited in GEO under accession number GSE171695. All published datasets analyzed in this study are detailed in Supplementary Data 2.

Field-specific reporting

Please select the one below that is the best fit for your research. If you are not sure, read the appropriate sections before making your selection.

☒ Life sciences ☐ Behavioural & social sciences ☐ Ecological, evolutionary & environmental sciences

For a reference copy of the document with all sections, see nature.com/documents/nr-reporting-summary-flat.pdf

Life sciences study design

All studies must disclose on these points even when the disclosure is negative.

Sample size	Sample sizes analyzed were chosen based on practical considerations and norms in the field for such genome-wide studies.
Data exclusions	Duplicated reads or multiple mapped reads were excluded from ChIP-seq analysis. This is standard practice for high-throughput sequencing datasets.
Replication	RNA-seq was carried out in duplicate for 48/50 samples and the data generated in all cases were consistent between the duplicates. For ChIP-seq, one replicate per condition was performed for all ChIP and input samples due to cost and availability of starting material at later differentiation stages. All datasets generated were included in our analyses.
Randomization	The experiments were not randomized. This study does not involve randomized samples.
Blinding	The investigators were not blinded to group allocation during sample collection or analysis. None of the analyses applied were based on subjective observations.

Reporting for specific materials, systems and methods

We require information from authors about some types of materials, experimental systems and methods used in many studies. Here, indicate whether each material, system or method listed is relevant to your study. If you are not sure if a list item applies to your research, read the appropriate section before selecting a response.

Materials & experimental systems

n/a	Involved in the study	
☐	☒	Antibodies
☐	☒	Eukaryotic cell lines
☒	☐	Palaeontology
☐	☒	Animals and other organisms
☒	☐	Human research participants
☒	☐	Clinical data

Methods

n/a	Involved in the study	
☐	☒	ChIP-seq
☐	☒	Flow cytometry
☒	☐	MRI-based neuroimaging

Antibodies

Antibodies used	ChIP-seq:<br>H3K9me3 (39161, Active Motif), H2AK119ub1 (8240, Cell Signaling)<br><br>Flow cytometry:<br>SSEA1-Phycoerythrin (PE) (125606, BioLegend), CD61-Alexa Fluor 647 (104314, BioLegend)
Validation	The histone mark antibodies and cell surface marker antibodies are widely used for ChIP-seq and flow cytometry, respectively. Validation statements of all antibodies are available on the manufacturers' websites (https://www.activemotif.com/catalog/details/39161; https://www.cellsignal.com/products/primary-antibodies/ubiquityl-histone-h2a-lys119-d27c4-xp-rabbit-

mab/8240; https://www.biolegend.com/en-us/products/pe-anti-mouse-human-cd15-ssea-1-antibody-4816?GroupID=GROUP20; https://www.biolegend.com/en-us/products/alexa-fluor-647-anti-mouse-rat-cd61-antibody-3325).

Eukaryotic cell lines

Policy information about cell lines

Cell line source(s)	Mouse embryonic stem cells (ESCs) were derived in the laboratory from E3.5 embryos.
Authentication	Cell lines were authenticated by genotyping.
Mycoplasma contamination	All cell lines were tested negative for mycoplasma contamination.
Commonly misidentified lines (See ICLAC register)	No misidentified cell lines were used in the study.

Animals and other organisms

Policy information about studies involving animals; ARRIVE guidelines recommended for reporting animal research

Laboratory animals	Mice used in this study were maintained on a C57BL/6 background. Primordial germ cells were isolated from embryonic day 9.5 male/female embryos.
Wild animals	This study did not involve wild animals.
Field-collected samples	This study did not involve samples collected from the field.
Ethics oversight	Mouse experiments were approved by the Animal Care Committee in the University of British Columbia under certificate numbers A16-0230, A16-0269, A20-0229 and A20-0230 with the guidelines from the national Canadian Council on Animal Care.

Note that full information on the approval of the study protocol must also be provided in the manuscript.

ChIP-seq

Data deposition

☒ Confirm that both raw and final processed data have been deposited in a public database such as GEO.

☒ Confirm that you have deposited or provided access to graph files (e.g. BED files) for the called peaks.

Data access links
May remain private before publication.

Raw and processed ChIP-seq and RNA-seq datasets are available at Gene Expression Omnibus (GEO) under accession number GSE171695.

Files in database submission

ChIP-seq:
Raw data:
H3K9me3_E9.5_PGC_1.fastq.bz2
H3K9me3_E9.5_PGC_2.fastq.bz2
H3K9me3_Mga_WT_nESC_1.fastq.bz2
H3K9me3_Mga_WT_nESC_2.fastq.bz2
H2AK119ub1_Mga_WT_nESC_1.fastq.bz2
H2AK119ub1_Mga_WT_nESC_2.fastq.bz2
Input_Mga_WT_nESC_1.fastq.bz2
Input_Mga_WT_nESC_2.fastq.bz2
H3K9me3_Mga_WT_EpiLC_1.fastq.bz2
H3K9me3_Mga_WT_EpiLC_2.fastq.bz2
H2AK119ub1_Mga_WT_EpiLC_1.fastq.bz2
H2AK119ub1_Mga_WT_EpiLC_2.fastq.bz2
Input_Mga_WT_EpiLC_1.fastq.bz2
Input_Mga_WT_EpiLC_2.fastq.bz2
H3K9me3_Mga_WT_d4PGCLC_1.fastq.bz2
H3K9me3_Mga_WT_d4PGCLC_2.fastq.bz2
H2AK119ub1_Mga_WT_d4PGCLC_1.fastq.bz2
H2AK119ub1_Mga_WT_d4PGCLC_2.fastq.bz2
Input_Mga_WT_d4PGCLC_1.fastq.bz2
Input_Mga_WT_d4PGCLC_2.fastq.bz2
H3K9me3_Mga_ΔHLH_nESC_1.fastq.bz2
H3K9me3_Mga_ΔHLH_nESC_2.fastq.bz2
H2AK119ub1_Mga_ΔHLH_nESC_1.fastq.bz2
H2AK119ub1_Mga_ΔHLH_nESC_2.fastq.bz2
Input_Mga_ΔHLH_nESC_1.fastq.bz2
Input_Mga_ΔHLH_nESC_2.fastq.bz2
H3K9me3_Mga_ΔHLH_EpiLC_1.fastq.bz2
H3K9me3_Mga_ΔHLH_EpiLC_2.fastq.bz2

H2AK119ub1_Mga_ΔHLH_EpiLC_1.fastq.bz2
H2AK119ub1_Mga_ΔHLH_EpiLC_2.fastq.bz2
Input_Mga_ΔHLH_EpiLC_1.fastq.bz2
Input_Mga_ΔHLH_EpiLC_2.fastq.bz2
H3K9me3_Setdb1_WT_nESC_1.fastq.bz2
H3K9me3_Setdb1_WT_nESC_2.fastq.bz2
H2AK119ub1_Setdb1_WT_nESC_1.fastq.bz2
H2AK119ub1_Setdb1_WT_nESC_2.fastq.bz2
Input_Setdb1_WT_nESC_1.fastq.bz2
Input_Setdb1_WT_nESC_2.fastq.bz2
H3K9me3_Setdb1_WT_EpiLC_1.fastq.bz2
H3K9me3_Setdb1_WT_EpiLC_2.fastq.bz2
H2AK119ub1_Setdb1_WT_EpiLC_1.fastq.bz2
H2AK119ub1_Setdb1_WT_EpiLC_2.fastq.bz2
H3K9me3_Setdb1_cKO_nESC_1.fastq.bz2
H3K9me3_Setdb1_cKO_nESC_2.fastq.bz2
H2AK119ub1_Setdb1_cKO_nESC_1.fastq.bz2
H2AK119ub1_Setdb1_cKO_nESC_2.fastq.bz2
Input_Setdb1_cKO_nESC_1.fastq.bz2
Input_Setdb1_cKO_nESC_2.fastq.bz2
H3K9me3_Setdb1_cKO_EpiLC_1.fastq.bz2
H3K9me3_Setdb1_cKO_EpiLC_2.fastq.bz2
H2AK119ub1_Setdb1_cKO_EpiLC_1.fastq.bz2
H2AK119ub1_Setdb1_cKO_EpiLC_2.fastq.bz2
H3K9me3_Pcgf6_WT_nESC_1.fastq.bz2
H3K9me3_Pcgf6_WT_nESC_2.fastq.bz2
H2AK119ub1_Pcgf6_WT_nESC_1.fastq.bz2
H2AK119ub1_Pcgf6_WT_nESC_2.fastq.bz2
Input_Pcgf6_WT_nESC_1.fastq.bz2
Input_Pcgf6_WT_nESC_2.fastq.bz2
H3K9me3_Pcgf6_WT_EpiLC_1.fastq.bz2
H3K9me3_Pcgf6_WT_EpiLC_2.fastq.bz2
H2AK119ub1_Pcgf6_WT_EpiLC_1.fastq.bz2
H2AK119ub1_Pcgf6_WT_EpiLC_2.fastq.bz2
Input_Pcgf6_WT_EpiLC_1.fastq.bz2
Input_Pcgf6_WT_EpiLC_2.fastq.bz2
H3K9me3_Pcgf6_cKO_nESC_1.fastq.bz2
H3K9me3_Pcgf6_cKO_nESC_2.fastq.bz2
H2AK119ub1_Pcgf6_cKO_nESC_1.fastq.bz2
H2AK119ub1_Pcgf6_cKO_nESC_2.fastq.bz2
Input_Pcgf6_cKO_nESC_1.fastq.bz2
Input_Pcgf6_cKO_nESC_2.fastq.bz2
H3K9me3_Pcgf6_cKO_EpiLC_1.fastq.bz2
H3K9me3_Pcgf6_cKO_EpiLC_2.fastq.bz2
H2AK119ub1_Pcgf6_cKO_EpiLC_1.fastq.bz2
H2AK119ub1_Pcgf6_cKO_EpiLC_2.fastq.bz2
Input_Pcgf6_cKO_EpiLC_1.fastq.bz2
Input_Pcgf6_cKO_EpiLC_2.fastq.bz2
H3K9me3_Dnmt_WT_nESC_1.fastq.bz2
H3K9me3_Dnmt_WT_nESC_2.fastq.bz2
H2AK119ub1_Dnmt_WT_nESC_1.fastq.bz2
H2AK119ub1_Dnmt_WT_nESC_2.fastq.bz2
Input_Dnmt_WT_nESC_1.fastq.bz2
Input_Dnmt_WT_nESC_2.fastq.bz2
H3K9me3_Dnmt_WT_EpiLC_1.fastq.bz2
H3K9me3_Dnmt_WT_EpiLC_2.fastq.bz2
H2AK119ub1_Dnmt_WT_EpiLC_1.fastq.bz2
H2AK119ub1_Dnmt_WT_EpiLC_2.fastq.bz2
Input_Dnmt_WT_EpiLC_1.fastq.bz2
Input_Dnmt_WT_EpiLC_2.fastq.bz2
H3K9me3_Dnmt_cTKO_nESC_1.fastq.bz2
H3K9me3_Dnmt_cTKO_nESC_2.fastq.bz2
H2AK119ub1_Dnmt_cTKO_nESC_1.fastq.bz2
H2AK119ub1_Dnmt_cTKO_nESC_2.fastq.bz2
Input_Dnmt_cTKO_nESC_1.fastq.bz2
Input_Dnmt_cTKO_nESC_2.fastq.bz2
H3K9me3_Dnmt_cTKO_EpiLC_1.fastq.bz2
H3K9me3_Dnmt_cTKO_EpiLC_2.fastq.bz2
H2AK119ub1_Dnmt_cTKO_EpiLC_1.fastq.bz2
H2AK119ub1_Dnmt_cTKO_EpiLC_2.fastq.bz2
Input_Dnmt_cTKO_EpiLC_1.fastq.bz2
Input_Dnmt_cTKO_EpiLC_2.fastq.bz2

Processed data:
H3K9me3_E9.5_PGC_w1000s100.RPM.bigWig

H3K9me3_Mga_WT_nESC_w1000s100.RPM.bigWig
H2AK119ub1_Mga_WT_nESC_w1000s100.RPM.bigWig
Input_Mga_WT_nESC_w1000s100.RPM.bigWig
H3K9me3_Mga_WT_EpiLC_w1000s100.RPM.bigWig
H2AK119ub1_Mga_WT_EpiLC_w1000s100.RPM.bigWig
Input_Mga_WT_EpiLC_w1000s100.RPM.bigWig
H3K9me3_Mga_WT_d4PGCLC_w1000s100.RPM.bigWig
H2AK119ub1_Mga_WT_d4PGCLC_w1000s100.RPM.bigWig
Input_Mga_WT_d4PGCLC_w1000s100.RPM.bigWig
H3K9me3_Mga_ΔHLH_nESC_w1000s100.RPM.bigWig
H2AK119ub1_Mga_ΔHLH_nESC_w1000s100.RPM.bigWig
Input_Mga_ΔHLH_nESC_w1000s100.RPM.bigWig
H3K9me3_Mga_ΔHLH_EpiLC_w1000s100.RPM.bigWig
H2AK119ub1_Mga_ΔHLH_EpiLC_w1000s100.RPM.bigWig
Input_Mga_ΔHLH_EpiLC_w1000s100.RPM.bigWig
H3K9me3_Setdb1_WT_nESC_w1000s100.RPM.bigWig
H2AK119ub1_Setdb1_WT_nESC_w1000s100.RPM.bigWig
Input_Setdb1_WT_nESC_w1000s100.RPM.bigWig
H3K9me3_Setdb1_WT_EpiLC_w1000s100.RPM.bigWig
H2AK119ub1_Setdb1_WT_EpiLC_w1000s100.RPM.bigWig
H3K9me3_Setdb1_cKO_nESC_w1000s100.RPM.bigWig
H2AK119ub1_Setdb1_cKO_nESC_w1000s100.RPM.bigWig
Input_Setdb1_cKO_nESC_w1000s100.RPM.bigWig
H3K9me3_Setdb1_cKO_EpiLC_w1000s100.RPM.bigWig
H2AK119ub1_Setdb1_cKO_EpiLC_w1000s100.RPM.bigWig
H3K9me3_Pcgf6_WT_nESC_w1000s100.RPM.bigWig
H2AK119ub1_Pcgf6_WT_nESC_w1000s100.RPM.bigWig
Input_Pcgf6_WT_nESC_w1000s100.RPM.bigWig
H3K9me3_Pcgf6_WT_EpiLC_w1000s100.RPM.bigWig
H2AK119ub1_Pcgf6_WT_EpiLC_w1000s100.RPM.bigWig
Input_Pcgf6_WT_EpiLC_w1000s100.RPM.bigWig
H3K9me3_Pcgf6_cKO_nESC_w1000s100.RPM.bigWig
H2AK119ub1_Pcgf6_cKO_nESC_w1000s100.RPM.bigWig
Input_Pcgf6_cKO_nESC_w1000s100.RPM.bigWig
H3K9me3_Pcgf6_cKO_EpiLC_w1000s100.RPM.bigWig
H2AK119ub1_Pcgf6_cKO_EpiLC_w1000s100.RPM.bigWig
Input_Pcgf6_cKO_EpiLC_w1000s100.RPM.bigWig
H3K9me3_Dnmt_WT_nESC_w1000s100.RPM.bigWig
H2AK119ub1_Dnmt_WT_nESC_w1000s100.RPM.bigWig
Input_Dnmt_WT_nESC_w1000s100.RPM.bigWig
H3K9me3_Dnmt_WT_EpiLC_w1000s100.RPM.bigWig
H2AK119ub1_Dnmt_WT_EpiLC_w1000s100.RPM.bigWig
Input_Dnmt_WT_EpiLC_w1000s100.RPM.bigWig
H3K9me3_Dnmt_cTKO_nESC_w1000s100.RPM.bigWig
H2AK119ub1_Dnmt_cTKO_nESC_w1000s100.RPM.bigWig
Input_Dnmt_cTKO_nESC_w1000s100.RPM.bigWig
H3K9me3_Dnmt_cTKO_EpiLC_w1000s100.RPM.bigWig
H2AK119ub1_Dnmt_cTKO_EpiLC_w1000s100.RPM.bigWig
Input_Dnmt_cTKO_EpiLC_w1000s100.RPM.bigWig

RNA-seq:
Raw data:
RNA_Dnmt_WT_nESC1_1.fastq.bz2
RNA_Dnmt_WT_nESC1_2.fastq.bz2
RNA_Dnmt_WT_nESC2_1.fastq.bz2
RNA_Dnmt_WT_nESC2_2.fastq.bz2
RNA_Dnmt_WT_EpiLC1_1.fastq.bz2
RNA_Dnmt_WT_EpiLC1_2.fastq.bz2
RNA_Dnmt_WT_EpiLC2_1.fastq.bz2
RNA_Dnmt_WT_EpiLC2_2.fastq.bz2
RNA_Dnmt_cTKO_nESC1_1.fastq.bz2
RNA_Dnmt_cTKO_nESC1_2.fastq.bz2
RNA_Dnmt_cTKO_nESC2_1.fastq.bz2
RNA_Dnmt_cTKO_nESC2_2.fastq.bz2
RNA_Dnmt_cTKO_EpiLC1_1.fastq.bz2
RNA_Dnmt_cTKO_EpiLC1_2.fastq.bz2
RNA_Dnmt_cTKO_EpiLC2_1.fastq.bz2
RNA_Dnmt_cTKO_EpiLC2_2.fastq.bz2
RNA_Mga_WT_nESC1_1.fastq.bz2
RNA_Mga_WT_nESC1_2.fastq.bz2
RNA_Mga_WT_nESC2_1.fastq.bz2
RNA_Mga_WT_nESC2_2.fastq.bz2
RNA_Mga_WT_EpiLC1_1.fastq.bz2
RNA_Mga_WT_EpiLC1_2.fastq.bz2

RNA_Mga_WT_EpiLC2_1.fastq.bz2
RNA_Mga_WT_EpiLC2_2.fastq.bz2
RNA_Mga_ΔHLH_nESC1_1.fastq.bz2
RNA_Mga_ΔHLH_nESC1_2.fastq.bz2
RNA_Mga_ΔHLH_nESC2_1.fastq.bz2
RNA_Mga_ΔHLH_nESC2_2.fastq.bz2
RNA_Mga_ΔHLH_EpiLC1_1.fastq.bz2
RNA_Mga_ΔHLH_EpiLC1_2.fastq.bz2
RNA_Mga_ΔHLH_EpiLC2_1.fastq.bz2
RNA_Mga_ΔHLH_EpiLC2_2.fastq.bz2
RNA_Setdb1_WT_nESC1_1.fastq.bz2
RNA_Setdb1_WT_nESC1_2.fastq.bz2
RNA_Setdb1_WT_nESC2_1.fastq.bz2
RNA_Setdb1_WT_nESC2_2.fastq.bz2
RNA_Setdb1_WT_EpiLC1_1.fastq.bz2
RNA_Setdb1_WT_EpiLC1_2.fastq.bz2
RNA_Setdb1_WT_EpiLC2_1.fastq.bz2
RNA_Setdb1_WT_EpiLC2_2.fastq.bz2
RNA_Setdb1_cKO_nESC1_1.fastq.bz2
RNA_Setdb1_cKO_nESC1_2.fastq.bz2
RNA_Setdb1_cKO_nESC2_1.fastq.bz2
RNA_Setdb1_cKO_nESC2_2.fastq.bz2
RNA_Setdb1_cKO_EpiLC1_1.fastq.bz2
RNA_Setdb1_cKO_EpiLC1_2.fastq.bz2
RNA_Setdb1_cKO_EpiLC2_1.fastq.bz2
RNA_Setdb1_cKO_EpiLC2_2.fastq.bz2
RNA_Pcgf6_WT_nESC1_1.fastq.bz2
RNA_Pcgf6_WT_nESC1_2.fastq.bz2
RNA_Pcgf6_WT_nESC2_1.fastq.bz2
RNA_Pcgf6_WT_nESC2_2.fastq.bz2
RNA_Pcgf6_WT_EpiLC1_1.fastq.bz2
RNA_Pcgf6_WT_EpiLC1_2.fastq.bz2
RNA_Pcgf6_WT_EpiLC2_1.fastq.bz2
RNA_Pcgf6_WT_EpiLC2_2.fastq.bz2
RNA_Pcgf6_cKO_nESC1_1.fastq.bz2
RNA_Pcgf6_cKO_nESC1_2.fastq.bz2
RNA_Pcgf6_cKO_nESC2_1.fastq.bz2
RNA_Pcgf6_cKO_nESC2_2.fastq.bz2
RNA_Pcgf6_cKO_EpiLC1_1.fastq.bz2
RNA_Pcgf6_cKO_EpiLC1_2.fastq.bz2
RNA_Pcgf6_cKO_EpiLC2_1.fastq.bz2
RNA_Pcgf6_cKO_EpiLC2_2.fastq.bz2
RNA_Ring1b_WT_pESC.fastq.gz
RNA_Ring1b_cKO_pESC.fastq.gz
RNA_Ring1b_WT_nESC1_1.fastq.bz2
RNA_Ring1b_WT_nESC1_2.fastq.bz2
RNA_Ring1b_WT_nESC2_1.fastq.bz2
RNA_Ring1b_WT_nESC2_2.fastq.bz2
RNA_Ring1b_WT_EpiLC1_1.fastq.bz2
RNA_Ring1b_WT_EpiLC1_2.fastq.bz2
RNA_Ring1b_WT_EpiLC2_1.fastq.bz2
RNA_Ring1b_WT_EpiLC2_2.fastq.bz2
RNA_Ring1b_cKO_nESC1_1.fastq.bz2
RNA_Ring1b_cKO_nESC1_2.fastq.bz2
RNA_Ring1b_cKO_nESC2_1.fastq.bz2
RNA_Ring1b_cKO_nESC2_2.fastq.bz2
RNA_Ring1b_cKO_EpiLC1_1.fastq.bz2
RNA_Ring1b_cKO_EpiLC1_2.fastq.bz2
RNA_Ring1b_cKO_EpiLC2_1.fastq.bz2
RNA_Ring1b_cKO_EpiLC2_2.fastq.bz2
RNA_Setdb1Ring1b_WT_nESC1_1.fastq.bz2
RNA_Setdb1Ring1b_WT_nESC1_2.fastq.bz2
RNA_Setdb1Ring1b_WT_nESC2_1.fastq.bz2
RNA_Setdb1Ring1b_WT_nESC2_2.fastq.bz2
RNA_Setdb1Ring1b_WT_EpiLC1_1.fastq.bz2
RNA_Setdb1Ring1b_WT_EpiLC1_2.fastq.bz2
RNA_Setdb1Ring1b_WT_EpiLC2_1.fastq.bz2
RNA_Setdb1Ring1b_WT_EpiLC2_2.fastq.bz2
RNA_Setdb1Ring1b_cDKO_nESC1_1.fastq.bz2
RNA_Setdb1Ring1b_cDKO_nESC1_2.fastq.bz2
RNA_Setdb1Ring1b_cDKO_nESC2_1.fastq.bz2
RNA_Setdb1Ring1b_cDKO_nESC2_2.fastq.bz2
RNA_Setdb1Ring1b_cDKO_EpiLC1_1.fastq.bz2
RNA_Setdb1Ring1b_cDKO_EpiLC1_2.fastq.bz2
RNA_Setdb1Ring1b_cDKO_EpiLC2_1.fastq.bz2

RNA_Setdb1Ring1b_cDKO_EpiLC2_2.fastq.bz2

Processed data:
RNA_Dnmt_WT_nESC1.RPM.bigWig
RNA_Dnmt_WT_nESC2.RPM.bigWig
RNA_Dnmt_WT_EpiLC1.RPM.bigWig
RNA_Dnmt_WT_EpiLC2.RPM.bigWig
RNA_Dnmt_cTKO_nESC1.RPM.bigWig
RNA_Dnmt_cTKO_nESC2.RPM.bigWig
RNA_Dnmt_cTKO_EpiLC1.RPM.bigWig
RNA_Dnmt_cTKO_EpiLC2.RPM.bigWig
RNA_Mga_WT_nESC1.RPM.bigWig
RNA_Mga_WT_nESC2.RPM.bigWig
RNA_Mga_WT_EpiLC1.RPM.bigWig
RNA_Mga_WT_EpiLC2.RPM.bigWig
RNA_Mga_ΔHLH_nESC1.RPM.bigWig
RNA_Mga_ΔHLH_nESC2.RPM.bigWig
RNA_Mga_ΔHLH_EpiLC1.RPM.bigWig
RNA_Mga_ΔHLH_EpiLC2.RPM.bigWig
RNA_Setdb1_WT_nESC1.RPM.bigWig
RNA_Setdb1_WT_nESC2.RPM.bigWig
RNA_Setdb1_WT_EpiLC1.RPM.bigWig
RNA_Setdb1_WT_EpiLC2.RPM.bigWig
RNA_Setdb1_cKO_nESC1.RPM.bigWig
RNA_Setdb1_cKO_nESC2.RPM.bigWig
RNA_Setdb1_cKO_EpiLC1.RPM.bigWig
RNA_Setdb1_cKO_EpiLC2.RPM.bigWig
RNA_Pcgf6_WT_nESC1.RPM.bigWig
RNA_Pcgf6_WT_nESC2.RPM.bigWig
RNA_Pcgf6_WT_EpiLC1.RPM.bigWig
RNA_Pcgf6_WT_EpiLC2.RPM.bigWig
RNA_Pcgf6_cKO_nESC1.RPM.bigWig
RNA_Pcgf6_cKO_nESC2.RPM.bigWig
RNA_Pcgf6_cKO_EpiLC1.RPM.bigWig
RNA_Pcgf6_cKO_EpiLC2.RPM.bigWig
RNA_Ring1b_WT_pESC.RPM.bigWig
RNA_Ring1b_cKO_pESC.RPM.bigWig
RNA_Ring1b_WT_nESC1.RPM.bigWig
RNA_Ring1b_WT_nESC2.RPM.bigWig
RNA_Ring1b_WT_EpiLC1.RPM.bigWig
RNA_Ring1b_WT_EpiLC2.RPM.bigWig
RNA_Ring1b_cKO_nESC1.RPM.bigWig
RNA_Ring1b_cKO_nESC2.RPM.bigWig
RNA_Ring1b_cKO_EpiLC1.RPM.bigWig
RNA_Ring1b_cKO_EpiLC2.RPM.bigWig
RNA_Setdb1Ring1b_WT_nESC1.RPM.bigWig
RNA_Setdb1Ring1b_WT_nESC2.RPM.bigWig
RNA_Setdb1Ring1b_WT_EpiLC1.RPM.bigWig
RNA_Setdb1Ring1b_WT_EpiLC2.RPM.bigWig
RNA_Setdb1Ring1b_cDKO_nESC1.RPM.bigWig
RNA_Setdb1Ring1b_cDKO_nESC2.RPM.bigWig
RNA_Setdb1Ring1b_cDKO_EpiLC1.RPM.bigWig
RNA_Setdb1Ring1b_cDKO_EpiLC2.RPM.bigWig

Genome browser session
(e.g. UCSC)

n/a

Methodology

Replicates

One replicate per condition was performed for all ChIP and input samples due to cost and availability of starting material at later differentiation stages. ChIP-seq was carried out on the parent line for each mutant line analyzed at each differentiation stage.

Sequencing depth

All data were sequenced as paired-end mode. Sequencing depth for read1 was shown below.

H3K9me3_E9.5_PGC_1.fastq.bz2 31,192,883
H3K9me3_Mga_WT_nESC_1.fastq.bz2 18,681,880
H2AK119ub1_Mga_WT_nESC_1.fastq.bz2 25,381,680
Input_Mga_WT_nESC_1.fastq.bz2 14,168,419
H3K9me3_Mga_WT_EpiLC_1.fastq.bz2 18,847,448
H2AK119ub1_Mga_WT_EpiLC_1.fastq.bz2 28,690,617
Input_Mga_WT_EpiLC_1.fastq.bz2 15,467,944
H3K9me3_Mga_WT_d4PGCLC_1.fastq.bz2 24,758,205
H2AK119ub1_Mga_WT_d4PGCLC_1.fastq.bz2 28,212,045
Input_Mga_WT_d4PGCLC_1.fastq.bz2 15,993,583

H3K9me3_Mga_ΔHLH_nESC_1.fastq.bz2 20,520,808
H2AK119ub1_Mga_ΔHLH_nESC_1.fastq.bz2 26,934,334
Input_Mga_ΔHLH_nESC_1.fastq.bz2 16,021,590
H3K9me3_Mga_ΔHLH_EpiLC_1.fastq.bz2 22,661,028
H2AK119ub1_Mga_ΔHLH_EpiLC_1.fastq.bz2 23,386,862
Input_Mga_ΔHLH_EpiLC_1.fastq.bz2 17,419,780
H3K9me3_Setdb1_WT_nESC_1.fastq.bz2 20,508,003
H2AK119ub1_Setdb1_WT_nESC_1.fastq.bz2 30,642,656
Input_Setdb1_WT_nESC_1.fastq.bz2 14,952,701
H3K9me3_Setdb1_WT_EpiLC_1.fastq.bz2 26,144,967
H2AK119ub1_Setdb1_WT_EpiLC_1.fastq.bz2 24,380,501
H3K9me3_Setdb1_cKO_nESC_1.fastq.bz2 20,395,579
H2AK119ub1_Setdb1_cKO_nESC_1.fastq.bz2 25,296,226
Input_Setdb1_cKO_nESC_1.fastq.bz2 23,149,750
H3K9me3_Setdb1_cKO_EpiLC_1.fastq.bz2 29,076,602
H2AK119ub1_Setdb1_cKO_EpiLC_1.fastq.bz2 27,745,615
H3K9me3_Pcgf6_WT_nESC_1.fastq.bz2 22,621,919
H2AK119ub1_Pcgf6_WT_nESC_1.fastq.bz2 27,158,870
Input_Pcgf6_WT_nESC_1.fastq.bz2 17,974,763
H3K9me3_Pcgf6_WT_EpiLC_1.fastq.bz2 19,665,937
H2AK119ub1_Pcgf6_WT_EpiLC_1.fastq.bz2 22,046,419
Input_Pcgf6_WT_EpiLC_1.fastq.bz2 8,301,355
H3K9me3_Pcgf6_cKO_nESC_1.fastq.bz2 20,916,955
H2AK119ub1_Pcgf6_cKO_nESC_1.fastq.bz2 24,506,570
Input_Pcgf6_cKO_nESC_1.fastq.bz2 15,557,462
H3K9me3_Pcgf6_cKO_EpiLC_1.fastq.bz2 16,609,242
H2AK119ub1_Pcgf6_cKO_EpiLC_1.fastq.bz2 28,996,051
Input_Pcgf6_cKO_EpiLC_1.fastq.bz2 13,207,891
H3K9me3_Dnmt_WT_nESC_1.fastq.bz2 28,197,844
H2AK119ub1_Dnmt_WT_nESC_1.fastq.bz2 61,745,494
Input_Dnmt_WT_nESC_1.fastq.bz2 8,276,308
H3K9me3_Dnmt_WT_EpiLC_1.fastq.bz2 25,007,173
H2AK119ub1_Dnmt_WT_EpiLC_1.fastq.bz2 46,889,738
Input_Dnmt_WT_EpiLC_1.fastq.bz2 9,371,181
H3K9me3_Dnmt_cTKO_nESC_1.fastq.bz2 24,670,093
H2AK119ub1_Dnmt_cTKO_nESC_1.fastq.bz2 38,316,852
Input_Dnmt_cTKO_nESC_1.fastq.bz2 8,313,656
H3K9me3_Dnmt_cTKO_EpiLC_1.fastq.bz2 35,620,991
H2AK119ub1_Dnmt_cTKO_EpiLC_1.fastq.bz2 50,151,993
Input_Dnmt_cTKO_EpiLC_1.fastq.bz2 9,865,414

Antibodies

H3K9me3 (39161, Active Motif), H2AK119ub1 (8240, Cell Signaling)

Peak calling parameters

Peak calling was not used for this study.

Data quality

The quality of the data was confirmed by their expected genomic annotations. Enrichment of histone marks were also validated by comparing with input signals.

Software

bedtools 2.22.1
bwa 0.7.10
IGV 2.3.98
picard-tools 1.128
R 4.0.0
samtools 1.1
VisR 0.9.41

Flow Cytometry

Plots

Confirm that:

☒ The axis labels state the marker and fluorochrome used (e.g. CD4-FITC).

☒ The axis scales are clearly visible. Include numbers along axes only for bottom left plot of group (a 'group' is an analysis of identical markers).

☒ All plots are contour plots with outliers or pseudocolor plots.

☒ A numerical value for number of cells or percentage (with statistics) is provided.

Methodology

Sample preparation

Details are described in the methods section.

Instrument	BD Influx
Software	BD FACS Diva
Cell population abundance	>90% of sorted PGCs was OCT4-EGFP+ and >95% of sorted PGCLCs was SSEA1-PE+/CD61-A647+ as assessed by resorting of sorted cells.
Gating strategy	FSC and SSC scatter plots were used to separate cell events from debris and/or dead cells.

☒ Tick this box to confirm that a figure exemplifying the gating strategy is provided in the Supplementary Information.
